# A Systematic Review of the Efficacy and Safety of Fecal Microbiota Transplantation in the Treatment of Hepatic Encephalopathy and Clostridioides difficile Infection in Patients With Cirrhosis

**DOI:** 10.7759/cureus.25537

**Published:** 2022-05-31

**Authors:** Kyaw Min Tun, Annie S Hong, Kavita Batra, Yassin Naga, Gordon Ohning

**Affiliations:** 1 Department of Internal Medicine, Kirk Kerkorian School of Medicine at the University of Nevada, Las Vegas, Las Vegas, USA; 2 Department of Gastroenterology and Hepatology, Kirk Kerkorian School of Medicine at the University of Nevada, Las Vegas, Las Vegas, USA; 3 Department of Research, Kirk Kerkorian School of Medicine at the University of Nevada, Las Vegas, Las Vegas, USA

**Keywords:** adverse outcomes, hepatic encephalopathy, clostridioides difficile infection, fecal microbiota transplantation, cirrhosis

## Abstract

The microbiome of the human gut and liver coexists by influencing the health and disease state of each system. Fecal microbiota transplantation (FMT) has recently emerged as a potential treatment for conditions associated with cirrhosis, such as hepatic encephalopathy and recurrent/refractory *Clostridioides difficile* infection (rCDI). We have conducted a systematic review of the safety and efficacy of FMT in treating hepatic encephalopathy and rCDI.

A literature search was performed using variations of the keywords “fecal microbiota transplant” and “cirrhosis” on PubMed/MEDLINE from inception to October 3, 2021. The resulting 116 articles were independently reviewed by two authors. Eight qualifying studies were included in the systematic review.

A total of 127 cirrhotic patients received FMT. Hepatic encephalopathy was evaluated by cognitive tests, such as the Psychometric Hepatic Encephalopathy Score (PHES) and EncephalApp Stroop test. Not only was there an improvement in the cognitive performance in the FMT cohort, but the improvement was also maintained throughout long-term follow-up. In the treatment of rCDI, the FMT success rate is similar between cirrhotic patients and the general population, although more than one dose may be needed in the former. The rate of serious adverse events and adverse events in the cirrhotic cohort was slightly higher than that in the general population but was low overall.

We found evidence that supports the therapeutic potential and safety profile of FMT to treat hepatic encephalopathy and rCDI in cirrhotic patients. Further research will be beneficial to better understand the role of FMT in cirrhosis.

## Introduction and background

Introduction

The human intestinal microbiome consists of trillions of microorganisms that colonize the entirety of the gastrointestinal tract. Recently, there have been reports in the literature that have highlighted the complex relationships between the intestinal microbiome and the health of the human body [1,2]. The gut microbiota has several functions. In addition to providing energy to the intestinal epithelium, the microbiome also regulates the intestinal barrier, the inflammatory cascade, the immune response, insulin resistance, and other functions [2]. Furthermore, as disruptions to the microbiota can alter a subpopulation species from a coexisting symbiote to a pathogen, balance among the subpopulations of the intestinal microbiome is crucial for the maintenance of health and prevention of disease [2].

The gut microbiome is susceptible to change due to external factors, such as alcohol use, dietary intake, medications, probiotics, and certain disease states [3,4]. In particular, there is emerging evidence that the quality and quantity of intestinal microbiota are intricately related to the health and illness of the liver, leading to the coining of the term “gut-liver axis” [3,5]. While the liver secretes bile acids into the intestines, it also functions as the first recipient and filter of the gut microbiota and the majority of nutrients and metabolites from the intestines through the portal vein [2,5]. Disruption to the balance of the subpopulations of the gut microbiome, also known as gut dysbiosis, can result in disruption of the intestinal barrier. Gut dysbiosis can impair the liver’s ability to differentiate harmful stimuli from harmless ones and can cause inappropriate activation of the inflammatory response [2,6]. On the other hand, it is hypothesized that factors often seen in chronic liver diseases (CLD), such as portal hypertension and immunological dysfunction, can disrupt the microbiome and lead to complications, such as translocation of bacteria and bacteria-derived byproducts, which can further worsen the liver injury, fibrosis, and systemic inflammation [2,3]. On a molecular level, sinusoidal cells in fibrotic livers produce less nitric oxide, a vasoactive agent, than in non-fibrotic livers [7,8]. Consequently, it can precipitate higher portal pressure that can lead to worsening of portal hypertension and subsequently to further complications, such as variceal bleeding.

Cirrhosis is the 11th leading cause of death worldwide and the third leading cause of death in individuals aged 45-64 years worldwide [8]. It is responsible for approximately one million deaths annually around the globe [8]. Cirrhosis has been associated with a myriad of complications, including but not limited to frequent hospitalizations, recurrent infections, hepatic encephalopathy, variceal hemorrhages, ascites, spontaneous bacterial peritonitis (SBP), and hepatorenal syndrome [8]. Among them, hepatic encephalopathy is the most common diagnosis for frequent admission to the hospital and places a burden on the patient, the caregiver, and the healthcare system [8]. Not only does hepatic encephalopathy lower the quality of life, but it also leads to increased mortality [9]. Research has shown that patients with cirrhosis and hepatic encephalopathy have altered composition from the native gut microbiome; lower populations of native taxa such as Lachnospiraceae and Ruminococcaceae and higher populations of Enterobacteriaceae and Firmicutes have been observed [9,10]. Currently, lactulose and rifaximin are the standards of care treatment for hepatic encephalopathy [11].

Due to frequent hospital admissions and antibiotic use, cirrhotic patients are at higher risk of developing *Clostridioides difficile* infection (CDI) [12,13]. More frequent incidents of complications from CDI have also been reported in patients with cirrhosis than in non-cirrhotic counterparts [10]. Furthermore, the frequent use of antibiotics due to infections such as peritonitis, urinary tract infections, and pneumonia in cirrhotic patients has been associated with multidrug-resistant (MDR) infections [1].

Significance of this study

Due to the significant and growing burden of cirrhosis on healthcare, different therapeutic modalities for the mitigation of CLD have been investigated in research trials, one of which is fecal microbiota transplantation (FMT). Although the pathophysiology is still unclear, research has demonstrated the complex relationship between the liver and the gut microbiome. While the use of FMT among cirrhotic patients has become more prominently discussed in the literature, there is limited published data and systematic reviews regarding the safety and efficacy of FMT in treating hepatic encephalopathy and recurrent and/or refractory *C. difficile* infection (rCDI). Furthermore, the scarcity of data from large research trials limits the use of FMT to often academic centers only. In the guidelines published in 2018 by the British Society of Gastroenterology (BSG) and Healthcare infectious Society (HIS), it was concluded that the role of FMT in cirrhosis patients is uncertain due to a lack of published evidence and consensus among multidisciplinary expert opinions [14,15]. Nevertheless, before embarking on large randomized controlled trials, the data from currently available literature must be studied and analyzed. Therefore, through this study, a systematic review was conducted to determine the efficacy and safety of FMT in the treatment of hepatic encephalopathy and rCDI among cirrhotic patients.

## Review

Methods

Search Strategy

A comprehensive literature search was performed using various keywords such as “fecal microbiota transplant” and “cirrhosis” including Medical Subject Headings (MeSH) terms to identify original studies published in PubMed/MEDLINE from inception to October 3, 2021. Results were limited to human studies published in English. There was a total of 116 studies available for review. Detailed search terms used to identify studies are available in the appendix section.

Eligibility Criteria

Inclusion criteria were studies with details on the following: (1) FMT for various indications, (2) established and detailed protocol for FMT administration, (3) patients with cirrhosis, (4) adult patients (≥18 years old), (5) reporting of outcomes after administration of the first FMT, (6) minimum follow-up period of eight weeks, and (7) at least moderate level of quality based on quality assessment tools as described below. A threshold of eight weeks was chosen based on the recommendations per European Society of Clinical Microbiology and Infectious Diseases (ESCMID) guidelines to monitor the response to FMT [16]. Exclusion criteria were (1) abstracts and review articles, (2) individual case reports, (3) non-English studies, (4) animal studies, and (5) patients younger than age 18.

Study Selection

Initially, 116 records were retrieved. After two authors (A.H. and K.T) independently reviewed the titles and abstracts, fourteen articles were determined to be relevant to our current study and were retrieved for full texts to be further reviewed. Afterward, eight studies fulfilled the eligibility criteria and the remaining six studies were excluded. If there was disagreement, a third reviewer (G.O.) was available for the final determination of whether a publication should be included in the systematic review. Institutional Review Board (IRB) approval was not required as the data were extracted from published studies. The selection process is described in the Preferred Reporting Items for Systematic Reviews and Meta-Analyses (PRISMA) diagram in the appendix figure.

Quality Assessment

Newcastle-Ottawa Scale (NOS) was used to evaluate the methodological quality of randomized controlled trials (RCT). The risk of bias regarding the selection of subjects, comparability of subjects, and assessment of the exposure and outcome was graded by using a star system corresponding to nine items. A study was categorized as low risk of bias if a total of 8 to 9 stars were allocated, medium risk of bias if 6 to 7 stars were allocated, and high risk of bias if the study was given ≤five stars [17]. A detailed assessment of the risk of bias in RCT is available in Appendix Table 6.

For case series, observational and cohort studies, appraisal of quality and risk of bias was performed by a series of quality assessment tools developed by the US National Heart Lung and Blood Institute (NHLBI) of the National Institutes of Health (NIH) (https://www.nhlbi.nih.gov/health-topics/study-quality-assessment-tools). Similar to NOS, a set of question items with Yes/No answers was used, with a “Yes” counting as a score of 1 and a “No” as a score of 0. In the tool used for the case series, there were a total of nine questions. A score of 7-9 corresponds to good quality, while scores of 4-6 and 1-3 indicate moderate and poor quality, respectively [17]. On the other hand, for observational and cohort studies, there were 14 items in total. However, three items were not applicable to the studies included in our systematic review. Out of the available 11 points, studies that score 7-11, 4-6, and 1-3 were graded as good, moderate, and poor quality, respectively [17]. In the final selection stage, only studies with at least a moderate level of evidence were included. Quality appraisal was performed by two independent authors (K.M.T. and K.B.). If there was any disagreement, a third reviewer (A.H.) evaluated the article and achieved consensus through discussion. Quality assessment of case series and observational cohort studies is available in Appendix Tables 7, 8, respectively.

Study Outcomes

The primary endpoints of the systematic review are the efficacy and safety of FMT in treating hepatic encephalopathy and *C. difficile* infection. In patients with hepatic encephalopathy, the efficacy is determined by the patient’s performance on cognitive tests, such as Psychometric Hepatic Encephalopathy Score (PHES) and EncephalApp Stroop test [18-20]. PHES is derived from five component tests: number connection test-A, number connection test-B, serial dotting test, line tracing test, and digit symbol test. It evaluates skills, such as attention, psychomotor function, and visual perception [21]. PHES has been validated as the gold standard for the diagnosis of hepatic encephalopathy as a patient’s performance on PHES has been demonstrated to be representative of his or her cerebral function [19,20]. A score that is mathematically lower than -4 is associated with hepatic encephalopathy and worse cognitive performance. EncephalApp Stroop test is a validated app-based version of the Stroop test. Similar to PHES, the Stroop test also evaluates cognitive flexibility and psychomotor speed [19]. It consists of two stages: the “ON” stage in which the participant names the color of the pound signs, and the “OFF” stage in which the participant states the color of a discordant word [18,20]. If the time taken for both ON and OFF stages is longer than 269.8 seconds, it has been determined that a diagnosis of hepatic encephalopathy can be made with sufficient sensitivity and specificity [18,20].

In cases with rCDI, the efficacy is determined by the resolution of diarrhea (<3 loose bowel movements in ≤24 hours) or other related symptoms, negative stool toxin test, no further need for antibiotics, and no recurrence of *C. difficile* within eight weeks from the first FMT [10,22]. The safety of FMT in patients with hepatic encephalopathy or rCDI is determined by the number of adverse events (AEs) and serious adverse events (SAEs) attributable to FMT following a certain period of the first administration. The follow-up period during which FMT can contribute to AEs and SAEs varies from study to study but is often eight to 12 weeks. SAEs are defined as events that led to unplanned emergency room visits, hospitalizations, life-threatening events, or death [10,18]. Other adverse effects that did not meet those criteria were labeled as AEs.

Data Extraction and Synthesis

As shown in the PRISMA diagram given in Appendix, data from eight studies were extracted by two authors (A.H. and K.M.T.) into customized data collection forms. Only studies with at least a moderate level of quality were included in the systematic review. After the quality assessment was performed [23], the following data were extracted from the studies: type of study, location of study, number of participants, etiology of cirrhosis, indication for FMT, method of FMT administration, and parameters for efficacy and safety. SAEs and AEs that were attributable to FMT per Data and Safety Monitoring Board (DSMB) guidelines were considered for the systematic review. Due to the inadequate number of studies, a meta-analysis was not performed.

Results

Search Results

An initial systematic search discovered 116 potentially relevant articles. Titles and abstracts of all 116 articles were reviewed; 102 articles were excluded for reasons such as animal studies (n = 7), non-cirrhotic patients (n = 5), pediatric patients (n = 1), case reports (n = 2), editorials (n = 5), review papers (n = 70), lack of FMT administration (n = 6), research protocols (n = 1), and systematic reviews (n = 5). Next, full texts of the remaining 14 papers were retrieved and evaluated for eligibility. Six articles were excluded due to being duplicate studies from the same population (n = 4) and lack of data on cirrhotic patients (n = 2). Eight studies were finally included in this review.

Study Characteristics

The included studies consisted of three RCTs, two retrospective studies, one prospective study, and two case series (Table 1). Most studies (5/8) were conducted in the United States. There was one study each from Spain and India. One study was based on data from multinational participants. The sample size varies from four patients in a case series to 272 participants in a prospective study.

**Table 1:** Summary of included studies FMT: Fecal microbiota transplant; RCT: Randomized controlled trial; USA: United States of America; NIH: National Institutes of Health; NOS: Newcastle-Ottawa Scale. *In this retrospective study by Meighani et al., a total of 201 patients were noted to have received FMT. Among them, there were 14 patients with chronic liver disease, nine of whom were identified to have cirrhosis. **In this prospective study by Pringle et al., 259 of 272 patients received FMT. Among those who were treated with FMT, 14 patients were diagnosed with cirrhosis.

Author/Year	Study Design	Quality Assessment	Quality Score	Location	Sample Size	Number of Patients who Received FMT	Follow-Up Period
Bajaj et al., 2021 [18]	RCT	NOS	9	Virginia, USA	20	10	6 months
Cheng et al., 2020 [10]	Retrospective study	NIH quality assessment tool	9	Multinational: USA, Canada, Italy	63	63	12 weeks
Meighani et al., 2020 [13]	Retrospective study	NIH quality assessment tool	8	Michigan, USA	201	9*	1 year
Pringle et al., 2019 [22]	Prospective study	NIH quality assessment tool	9	Massachusetts, USA	272	14**	8 weeks
Bajaj et al., 2019 [24]	RCT	NOS	8	Virginia, USA	20	10	6 months
Bajaj et al., 2019 [6]	RCT	NOS	8	Virginia, USA	13	7	12-15 months
Mehta et al., 2018 [25]	Case series	NIH quality assessment tool	7	Surat, India	10	10	20 weeks
Olmedo et al., 2019 [26]	Case series	NIH quality assessment tool	8	Madrid, Spain	4	4	4-11 months

The route of FMT administration also varied. While some studies only used one type of route, multiple methods of delivering FMT were used in others. Overall, the methods included enema (3), capsule (3), colonoscopy (4), a nasogastric tube (2), and percutaneous endoscopic gastrostomy (1) (Tables 2, 3). Only patients with documented cirrhosis who received FMT were included in the systematic review. As such, from the combined total of 603 patients, 127 participants received FMT.

**Table 2:** Summary of publications that studied the utilization of FMT in the treatment of hepatic encephalopathy in cirrhotic patients FMT: Fecal microbiota transplant; AEs: Adverse events; SAEs: Serious adverse events; PHES: Psychometric Hepatic Encephalopathy Score; SBP: Spontaneous bacterial peritonitis; PO: per os (by mouth); BID: bis in die (twice daily); TID: ter in die (three times daily). *Interquartile range (IQR). **Mean ± Standard deviation. ^¥^Median (range). ^£^Median and 95% confidence interval: Data were extracted from a bar chart using a digitizer software and rounded to one decimal place. The data reported in the table is from a one-year follow-up. ^ƍ^Mehta et al. evaluated the efficacy of FMT based on the recurrence of overt hepatic encephalopathy or readmission to the hospital during the 20-week follow-up. Six adverse events occurred among four patients. Three patients continued to have overt hepatic encephalopathy. Two were treated on an outpatient basis; one expired due to sepsis from bronchopneumonia. There were two readmissions to the hospital due to SBP, each of which was respectively caused by *Streptococcus gallolyticus* subspecies *pasteurianus* and *Enterococcus fecalis*.

Author/Year	Type of Donor	Method of FMT Administration	Exposure to Antibiotics Prior to FMT	Cognitive Performance Before FMT	Cognitive After FMT	Number of AEs	Number of SAEs
Bajaj et al., 2021 [18]	Single donor	Enema	None	PHES: -5.5 (-10.00-0.0)*; EncephalApp: 197.8 (164.7-222.1)*	PHES: -2.5 (-9.25-1.00)*; EncephalApp: 187.5 (167.8-213.3)*	Not reported	0 [0.25]*
Bajaj et al., 2019 [24]	Single donor enriched in* Lachnospiraceae* and *Ruminococcaceae*	Capsule	None (all subjects were on rifaximin)	PHES: -6.8 ± 6.3**; EncephalApp: 277.8 ± 123.5	PHES: -5.7 ± 5.4; EncephalApp: 226.7 ± 56.1	Constipation (n = 2); diarrhea (n = 1); bloating (n = 1)	1 (0,0-1)^¥^ : a case of hepatic encephalopathy that was found to be unrelated to FMT
Bajaj et al., 2019 [6]	Single donor	Enema	Ciprofloxacin 500 mg PO BID, Amoxicillin 500 mg PO TID, Metronidazole 500 mg PO TID for 5 days	PHES: -6.5 (-9.9, -3.9)^£^; EncephalApp: 237.2 (218.1, 271.9)^£^	PHES: -5.9 (-7.9, -3.9)^ £^; EncephalApp: 222.2 (203.1, 232.9)^£^	Not reported	Ascites (n = 1)
Mehta et al., 2018 [25]	Patient-identified	Colonoscopy	All patients were given broad-spectrum antibiotics for 5 days	Not utilized^ƍ^	Not utilized^ƍ^	Recurrence of hepatic encephalopathy (n = 2)^&^	Death due to sepsis from bronchopneumonia(n = 1)^&^; hepatic encephalopathy (n = 1)^&^; SBP (n = 2)^&^

**Table 3:** Summary of publications that studied the utilization of FMT in the treatment of Clostridioides difficile infection in cirrhotic patients FMT: Fecal microbiota transplant; AEs: Adverse events; SAEs: Serious adverse events; SBP: Spontaneous bacterial peritonitis; CDI: *Clostridioides difficile* infection; *E. coli*: *Escherichia coli*. *Among the patients who received rifaximin and antibiotics for SBP prophylaxis, three and two patients failed FMT, respectively. ^ƍ^All 14 patients were treated with oral vancomycin before being referred for FMT. Additional antibiotic exposure was noted in three patients. **While it is known that nine of 14 CLD patients were cirrhotic, the success or failure rate was not stratified for cirrhosis vs non-cirrhosis patients or recent antibiotic exposure.

Author/Year	Type of Donor	Method of FMT Administration	Exposure to Antibiotics Prior to FMT	Number of Patients With FMT Success	Number of Patients With FMT Failure	Number of AEs	Number of SAEs
Cheng et al., 2020 [10]	Patient-directed (n = 3); Universal donor (n = 35); Stool bank (n = 25)	Capsule (n = 3); Colonoscopy (n = 59); Percutaneous endoscopic gastrostomy (n = 1)	15 (Rifaximin); 6 (SBP prophylaxis)	54/63 (85%: 48 of 55 for recurrent CDI and 6 of 8 for severe or fulminant CDI)	9/63 (14%: 7 of 55 for recurrent CDI and 2 of 8 for severe or fulminant CDI)*	Abdominal pain/cramping (n = 10); diarrhea (n = 9)	5 (Crohn’s disease flare, fecal urgency, acute kidney injury postprocedure, hepatic encephalopathy, portal hypertensive bleed)
Meighani et al., 2020 [13]	Family members or universal FMT donors	Colonoscopy (n = 5); Enema (n = 2); Nasogastric tube (n = 7)	3^ƍ^	12/14 (87%)**	2/14**	Not reported	Not reported
Pringle et al., 2019 [22]	Unrelated healthy donors of age 18-49	Capsule	Not reported	13/14 (reported as 92.9% of cirrhotic patients)	1/14	Not reported	Not reported
Olmedo et al., 2019 [26]	Unknown	Colonoscopy (n = 3); Nasogastric tube (n = 1)	Metronidazole, Vancomycin tapering, Fidaxomicin	3/4 (75%)	1/4	Not reported	*E. coli* bacteremia (n = 1); death due to cholangitis (n = 1)

Results of studies using FMT in hepatic encephalopathy are reported in Table 2. The PHES and EncephalApp Stroop tests were used as a measure of hepatic encephalopathy in most studies. A mathematically higher score on PHES is associated with better cognitive performance. On the other hand, a longer duration (in seconds) on EncephelApp Stroop test score suggests poorer performance [24].

Performance on cognitive measurement was not reported in Mehta et al. [25]. Instead, recurrence of hepatic encephalopathy was documented as a measure of the efficacy of FMT. By week 20, most patients who received FMT had no recurrence of hepatic encephalopathy. In the study by Bajaj et al. in 2019 [6], the data was presented as a bar chart. The numerical values were extracted using a web plot digitizer software, rounded to one decimal place, and reported in Table 2. Overall, across all studies, clinically significant improvement in performance on cognitive tests was noted in the treatment cohort.

The rate of success and outcomes for FMT in cirrhotic patients with CDI are reported in Table 3. There was no significant difference in baseline characteristics or route of FMT delivery among cirrhotic patients who failed or achieved cure with FMT. However, Cheng et al. reported that severity of cirrhosis, such as Child-Pugh Class B or C, or severity of CDI, such as the presence of pseudomembranous colitis, was associated with a higher incidence of FMT failures [9,10]. Child-Pugh Class B/C was seen in 100% of patients who had FMT failure (vs 37.7% of patients who had FMT success), and pseudomembranous colitis was present in 22.2% of FMT failures and 0% of FMT successes [10]. Additionally, while FMT was found to be effective in the treatment of CDI, Pringle et al. reported that cirrhotic patients required more than one dose of FMT to achieve cure compared to their non-cirrhotic counterparts [22].

Baseline characteristics and disease severity were also associated with the success and failure rates of FMT. Cirrhosis itself puts patients at a higher probability of requiring multiple doses of FMT to cure CDI (odds ratio: 10.65; 95% CI: 3.31-34.27; p < 0.0001) [22]. Pringle et al. concluded that more individuals with cirrhosis required two to three doses of FMT in lieu of one dose compared to the non-cirrhotic cohort (22.6% vs 2.7%, p < 0.0001) to achieve a cure rate, with an odds ratio of 18.24 (95% CI: 3.18-104.89, p = 0.001) [22]. Furthermore, pre-FMT stage of cirrhosis is also associated with an increased risk of FMT failure. In the retrospective study by Meighani et al., both patients who did not respond to FMT had decompensated cirrhosis with Child-Turcotte-Pugh scores of 9 and 12 [13]. Similarly, in a retrospective study by Cheng et al., 100% of FMT failures were seen in patients with Child-Pugh B or C stage of cirrhosis (p < 0.001) [10]. Similarly, Pringle, et al. found that patients with higher MELD scores required more than one dose of FMT [22]. The presence of pseudomembranous colitis on endoscopy also corresponded to higher rates of FMT failure [10].

There were 12 SAEs and 25 AEs compiled from a total of 127 cirrhotic patients across all studies included in the systematic review. Among the SAEs, recurrence of hepatic encephalopathy was the most common and occurred in three patients, one of whom later developed bronchopneumonia and subsequently expired. There was one case of hepatic encephalopathy reported by Bajaj et al. that was found to be unrelated to FMT but was a complication of transjugular intrahepatic portosystemic shunt [24]. Among the two deaths observed, the other etiology of mortality was cholangitis. There was no other death reported to be related to FMT. Other events requiring hospitalization, emergency room visits, or death included two cases of SBP, one case of ascites, and one case of bacteremia (Table 4). Diarrhea and abdominal cramping were the two most common AEs (Table 5).

**Table 4:** Etiology and frequency of serious adverse events *One patient with hepatic encephalopathy later expired as a result of sepsis from bronchopneumonia. The said patient was counted separately for both hepatic encephalopathy and death.

Serious Adverse Events	Frequency
Death	2
Hepatic encephalopathy	3*
Spontaneous bacterial peritonitis	2
Ascites	1
Crohn’s disease flare	1
Fecal urgency	1
Acute kidney injury	1
Portal hypertensive bleed	1
*E. coli* bacteremia	1

**Table 5:** Etiology and frequency of adverse events

Adverse Events	Frequency
Constipation	2
Diarrhea	10
Bloating	1
Recurrence of hepatic encephalopathy	2
Abdominal pain and cramping	10

Discussion

Use of FMT in the Treatment of Hepatic Encephalopathy

As reported above, FMT is shown to be effective in treating hepatic encephalopathy. An improvement in cognitive tests (PHES and EncephalApp Stroop test) or reduced recurrence of hepatic encephalopathy was seen across all the studies. Furthermore, the effect of FMT was maintained over the course of the year. Bajaj et al. in 2019 reported improved scores on the cognitive tests on day 5 or day 10 after receiving FMT. In the long-term follow-up of the same participants, the cognitive performance remained superior to the baseline data from pre-FMT administration (Appendix Table 9) [6]. Moreover, fewer episodes of hepatic encephalopathy and hospitalizations were noted in the cohort treated with FMT compared to the control group [6]. Hence, not only does FMT improve the immediate cognitive outcome, but the effect is also maintained over the course of a year. Previously, one of the factors that prevent more robust use of FMT in cirrhotic patients is a concern for worsening of hepatic encephalopathy [9].

Nonetheless, this systematic review suggests that FMT is in fact effective in treating and lowering the recurrence of hepatic encephalopathy. Moreover, the level of arterial ammonia also decreased significantly following the treatment of FMT. The ammonia concentration was 96 (87.25-117.75), whereas the level declined to 74 (70-82) at week 20 post-FMT in the case series by Mehta et al. [25]. From the same study, a statistically significant reduction in liver parameters such as the Child-Turcotte-Pugh score (from 9.5 [9-10.75] to 8 [7-8]) and MELD score (from 18 [16.25-19] to 15 [14-16]) was also observed [25]. Therefore, the evidence suggests that FMT may be beneficial in hindering the progression of chronic liver disease.

Among the AEs and SAEs reported, only those considered to be associated with FMT per DSMB guidelines were included in the systematic review. It must be noted that complications such as ascites, SBP, and hepatic encephalopathy are common in this population. While the adverse outcomes may not be a direct consequence of FMT, we cannot definitively exclude FMT from one of the associated factors. Nevertheless, in the long-term follow-up of patients by Bajaj et al., a higher number of SAEs and AEs were noted in the standard-of-care (SOC) cohort compared to the FMT cohort [6]. For instance, there were 10 hospitalizations in the SOC arm and one hospitalization in the FMT arm [6]. Therefore, FMT has been found to achieve a higher rate of success and a lower rate of unfavorable results compared to SOC.

Use of FMT in the Treatment of rCDI

FMT appears to be an effective treatment for the rCDI in cirrhotic patients with similar outcomes compared with other populations [9,10,22]. The cure rate was observed between 75% and 92% among the studies included in our systematic review. Previously, a systematic review by Shogbesan et al. established that the FMT success rate was similar between immunocompromised and immunocompetent patients [27]. FMT success rate was 88% after a single FMT and 93% after multiple FMTs in the immunocompromised cohort, whereas it was 80%-90% in the reference cohort [27]. Nevertheless, patients with a single immunocompromising factor achieved a higher rate of treatment success than those with multiple immunocompromising factors [27]. Furthermore, immunodeficient patients required more than one dose of FMT to achieve a cure rate [27]. Similarly, because of the dysregulated immune system, patients with cirrhosis are at higher risk of developing CDI and rCDI even in the absence of recent antibiotic exposure [9]. Hence, although FMT has been shown to be an effective treatment for rCDI in a cirrhotic population, a patient’s immune status may dictate the number of doses required to achieve resolution. Our systematic review also discovered that patients with cirrhosis did not result in worsening the Child-Pugh Turcotte score after the administration of FMT [9].

Only Cheng et al. reported the number of AEs. The rate of AEs among the cirrhotic cohort (30.2%) was comparable to the rate reported by a 2016 systematic review by Wang et al. that included all patients (28.5%) [10]. Overall the rate of complications observed among patients with cirrhosis who received FMT was low: 21% and 8%, respectively, for AEs and SAEs. Olmedo et al. reported a case of a patient who developed cholangitis and died within seven days of FMT; blood cultures had not been obtained at the time of expiration [26]. It was noted that the patient had a history of recurrent cholangitis.

These results are comparable to those from other populations. A systematic review was performed by Shogbesan et al. in 2018 regarding the use of FMT to treat CDI in immunocompromised patients such as those with malignancy, human immunodeficiency virus (HIV), immunodeficiency syndromes, a history of organ transplant, or those who are on immunosuppressants [27]. Rehospitalization was noted in 8.3% of patients and death in two patients (approximately 0.6% of patients) [26]. The success and SAE rates were similar to those reported in our systematic review. A separate study was conducted by Youngster et al. regarding the use of FMT to treat rCDI in the general population. The resolution was observed in 70% and 95% of 20 patients after the first and multiple treatments, respectively [28,29]. There were no SAEs; AEs were reported in 30% of patients [29]. While it is a single-center study, there was a relatively lower number of complications in immunocompetent patients when compared to cirrhotic and immunocompromised patients. However, our systematic review demonstrates that SAEs and AEs remain uncommon in the latter group. The cure rate in the general population from Youngster et al. and in the cirrhotic population from our systematic review is comparable.

Other Outcomes Associated With FMT

In addition to lowering the recurrence of hepatic encephalopathy and CDI, FMT is also associated with other beneficial outcomes. In a study by Bajaj et al. in 2020, the impact of FMT on alcohol use disorder is also assessed by the alcohol craving questionnaire-short form (ACQ-SF), which is a series of 12 questions that is graded on a scale of 1 to 7 [18]. A higher ACQ-SF score is suggestive of higher craving [18]. Patients from the FMT cohort had lower scores on ACQ-SF questionnaire, indicative of reduced craving and alcohol consumption [18]. From the study by Bajaj et al., the median and interquartile range of ACQ-SF score was 3.1 (2.4-4.5) pre-treatment, which improved to 2.5 (2.1-4.3) post-treatment [18]. It has been demonstrated that certain patterns of gut microbiota are affiliated with impaired inhibitory control seen in addictive disorders [30]. Given this information, it is possible that when alterations in the gut microbiome occur through FMT, the corresponding maladaptive behaviors are modified as well.

Apart from possibly discouraging addictive behaviors, FMT also leads to an improvement in the psychosocial aspect of the quality of life reported by measuring sickness impact profile (SIP), which evaluates a patient’s perception of their own health status [18]. In addition, lower serum concentrations of inflammatory markers such as interleukin-6 (IL-6) and lipopolysaccharide-binding protein (LBP) were noted [18]. On the other hand, there was a statistically significant increase in defensin A5, which is a different type of inflammatory marker and is a part of antimicrobial peptides [4].

Other potential benefits of FMT include an increase in insulin sensitivity in individuals with metabolic syndrome or obesity [9]. FMT can also be used to decolonize multidrug-resistant organisms along the gastrointestinal tract and can potentially help reduce complication rates and improve health outcomes [31]. These findings, however, were not exclusive to cirrhotic subjects [9,31].

One of the factors that had been shown to have a statistically significant correlation with the rate of FMT failure was prior exposure to antimicrobials and probiotics [10]. Based on the results from the general population, Fischer et al. concluded that the use of non-CDI antibiotics within eight weeks of FMT was one of the variables associated with early FMT failure [32]. A similar result was discovered in the retrospective study by Cheng et al. based on data from cirrhotic patients [10]. Rifampin and antibiotics for SBP prophylaxis were not considered non-CDI antibiotics. In the study by Meighani et al., where all patients were treated with oral vancomycin at baseline, three patients were also noted to have been exposed to antibiotics besides vancomycin; however, the results were not stratified for the said patients [13].

On the other hand, the use of probiotics has also been linked to FMT failure. In the study by Cheng et al., 77.8% of patients who failed FMT were found to have used probiotics prior to FMT [10]. In comparison, 24.1% of patients who achieved FMT success used probiotics [10]. Cheng et al. reported that using probiotics immediately before FMT increased the risk of FMT failure almost as high as 12-fold (odds ratio: 11.9; 95% CI: 1.81-78.3, p = 0.01) [10]. Interestingly, the use of probiotics or non-CDI antibiotics immediately after successful FMT has also been associated with an increased risk of recurrence of CDI in a study by Allegretti et al. [33]. It is believed that probiotics delay or interrupt the recovery of the gut microbiota [32-34].

Study limitations

There are several limitations in our systematic review. First, only articles published in English were included, which could have potentially excluded relevant studies that were published in a different language. Second, there were AEs and SAEs that were reported in the original studies but were considered unrelated to FMT by the original authors based on the DSMB guidelines. Since we were not able to obtain the original data of the studies, it is possible that the number of AEs attributable to FMT may be inaccurate. Third, due to the small number of studies available, a meta-analysis could not be performed. Next, information about the health status of the donor and the sourcing of donor material was not described in detail in all the included studies and maybe a potential confounding factor. Lastly, only PubMed/MEDLINE was used as it is the largest and most robust database, which may not have extracted smaller studies and may have introduced publication bias.

## Conclusions

This study aims to better understand the emerging therapeutic potential of FMT in cirrhotic patients by focusing on the performance of cognitive tests, recurrence of CDI, and adverse outcomes. Our systematic review of currently published literature suggests that there is therapeutic efficacy of FMT in treating hepatic encephalopathy and rCDI in cirrhotic patients. It was discussed that the stage of cirrhosis, the severity of CDI, and the use of probiotics or antibiotics may impair the effectiveness of FMT. Our review found that while AEs from FMT seem to be slightly higher than those from the general population, negative outcomes appear to be comparable to the rate of complications from other similarly immunocompromised populations. Nevertheless, the rate of SAEs and AEs in cirrhotic patients remains low, especially when compared to the potential benefits. Therefore, based on the available data, we conclude that FMT should be considered a safe and effective treatment option in patients with cirrhosis. However, more research trials with larger cohorts and robust study protocols are needed to further elucidate the role of FMT in cirrhotic patients.

## Figures and Tables

**Figure 1:**
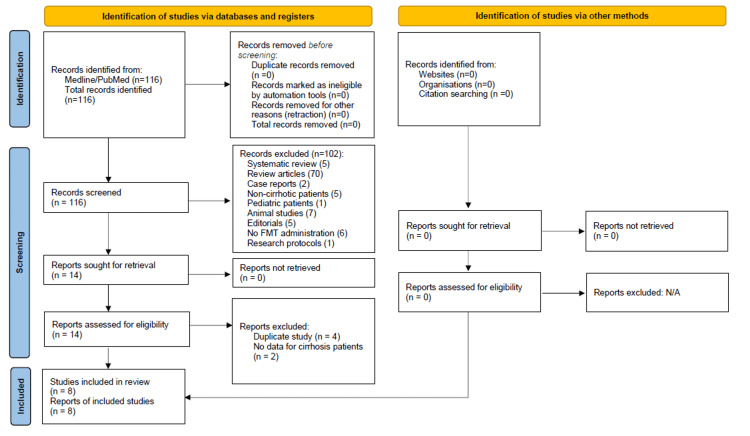
PRISMA (Preferred Reporting Items for Systematic Reviews and Meta-Analyses) diagram of the study selection and data extraction process FMT: Fecal microbiota transplantation.

## Appendices

Search terms used to identify and filter studies from the literature

Keywords: Fecal microbiota transplant cirrhosis.

Inclusion: Any type of study with patient data and outcomes, any age, and any sex.

Exclusion: Animal studies and single case reports.

Decompensated cirrhosis: CPC

PubMed

("fecal microbiota transplantation" OR "faecal microbiota transplantation" OR "fecal microbiota transplant" OR "faecal microbiota transplant" OR "fecal microbiota transfer" OR "faecal microbiota transfer" OR "fecal transplant" OR "faecal transplant" OR "fecal transfer" OR "faecal transfer" OR "donor feces" OR "donor faeces" OR "donor stool" OR "bacteriotherapy" OR "FMT" OR "Fecal Microbiota Transplantation"[mh]) AND ("cirrhosis" OR "liver disease" OR "liver cirrhosis"[mh]) NOT animal.

Filter: All studies

Filter: English

**Table 6:** Risk of bias assessment of randomized controlled trials BMI: Body mass index.

Study	Acceptable (*)	Bajaj et al., 2021 [18]	Bajaj et al., 2019 [24]	Bajaj et al., 2019 [6]
Selection				
1. Is the case definition adequate?	Yes, with independent validation	*	*	*
2. Representativeness of the cases	Consecutive or obviously representative series of cases	*	*	*
3. Selection of controls	Community controls	*	*	*
4. Definition of controls	Did not receive intervention	*	*	*
Comparability				
5. Study controls for age/sex	Yes	*	*	*
6. Study controls for at least three additional factors	BMI, ethnicity, family history, smoking, alcohol, physical activity, diet, diabetes mellitus	*	-	*
Exposure				
7. Ascertainment of exposure	Secure record; structured interview by a healthcare practitioner; blinded to case/control status	*	*	*
8. Same method of ascertainment for cases and controls	Yes	*	*	*
9. Non-response rate	Same rate for both groups	*	*	-
Total quality score	Total number of *	9	8	8
Quality (Score ≥ 7: high-quality study)		Good	Good	Good

**Table 7:** Risk of bias assessment of case series Y: Yes; N: No.

Study	Mehta et al., 2018 [25]	Olmedo et al., 2019 [26]
1. Was the study question or objective clearly stated?	Y	Y
2. Was the study population clearly and fully described, including a case definition?	Y	Y
3. Were the cases consecutive?	Y	Y
4. Were the subjects comparable?	N	N
5. Was the intervention clearly described?	Y	Y
6. Were the outcome measures clearly defined, valid, reliable, and implemented consistently across all study participants?	Y	Y
7. Was the length of follow-up adequate?	Y	Y
8. Were the statistical methods well-described?	N	Y
9. Were the results well-described?	Y	Y
Total quality score (total number of “yes”)	7	8
Quality (7-9 yes: Good; 4-6 yes: Fair; 1-3 yes: Poor)	Good	Good

**Table 8:** Risk of bias assessment of observational cohort studies Y: Yes; N: No.

Study	Cheng et al., 2021 [10]	Meighani et al., 2020 [13]	Pringle et al., 2019 [22]
1. Was the study question or objective clearly stated?	Y	Y	Y
2. Was the study population clearly and fully described?	Y	Y	Y
3. Was the participation rate of eligible persons at least 50%?	Not applicable	Not applicable	Not applicable
4. Were all the subjects selected or recruited from the same or similar populations (including the same time period)? Were inclusion and exclusion criteria for being in the study prespecified and applied uniformly to all participants?	Y	Y	Y
5. Was a sample size justification, power description, or variance and effect estimates provided?	N	N	N
6. For the analyses in this paper, were the exposure(s) of interest measured prior to the outcome(s) being measured?	Y	Y	Y
7. Was the timeframe sufficient so that one could reasonably expect to see an association between exposure and outcome if it existed?	Y	Y	Y
8. For exposures that can vary in amount or level, did the study examine different levels of exposure as related to the outcome (e.g., categories of exposure or exposure measured as a continuous variable)?	Y	N	N
9. Were the exposure measures (independent variables) clearly defined, valid, reliable, and implemented consistently across all study participants?	Y	Y	Y
10. Was the exposure(s) assessed more than once over time?	N	N	Y
11. Were the outcome measures (dependent variables) clearly defined, valid, reliable, and implemented consistently across all study participants?	Y	Y	Y
12. Were the outcome assessors blinded to the exposure status of participants?	Not applicable	Not applicable	Not applicable
13. Was the loss to follow-up after baseline 20% or less?	Not applicable	Not applicable	Not applicable
14. Were key potential confounding variables measured and adjusted statistically for their impact on the relationship between exposure(s) and outcome(s)?	Y	Y	Y
Total quality score (total number of “yes”)	9	8	9
Quality (7-9 yes: Good; 4-6 yes: Fair; 1-3 yes: Poor)	Good	Good	Good

**Table 9:** Cognitive test results at different points in time based on long-term follow up of participants from Bajaj et al., 2019 The data were reported as a bar chart in the original paper by Bajaj et al. [6]. The numerical values were extracted using a web plot digitizer software and were rounded to one decimal place. It was reported as a median and 95% confidence interval. PHES: Psychometric Hepatic Encephalopathy Score; FMT: Fecal microbiota transplantation.

	PHES	EncephalApp Stroop test
Baseline	-6.5 (-9.9,-3.9)	237.2 (218.1,271.9)
Day 5 Post-FMT	-3.9 (-8.9,-3.7)	219.3 (198.4,249.7)
Day 10 Post-FMT	-3.9 (-7.7,-3.0)	208.5 (177.5,228.8)
One year post-FMT	-5.9 (-7.9,-3.9)	222.2 (203.1,232.9)

## References

[1] Bajaj JS, Shamsaddini A, Fagan A (2021). Fecal microbiota transplant in cirrhosis reduces gut microbial antibiotic resistance genes: analysis of two trials. Hepatol Commun.

[2] Albhaisi SA, Bajaj JS, Sanyal AJ (2020). Role of gut microbiota in liver disease. Am J Physiol Gastrointest Liver Physiol.

[3] Arab JP, Martin-Mateos RM, Shah VH (2018). Gut-liver axis, cirrhosis and portal hypertension: the chicken and the egg. Hepatol Int.

[4] Madsen M, Kimer N, Bendtsen F, Petersen AM (2021). Fecal microbiota transplantation in hepatic encephalopathy: a systematic review. Scand J Gastroenterol.

[5] Haque TR, Barritt AS 4th (2016). Intestinal microbiota in liver disease. Best Pract Res Clin Gastroenterol.

[6] Bajaj JS, Fagan A, Gavis EA, Kassam Z, Sikaroodi M, Gillevet PM (2019). Long-term outcomes of fecal microbiota transplantation in patients with cirrhosis. Gastroenterology.

[7] Chen B, Huang H, Pan CQ (2022). The role of gut microbiota in hepatitis B disease progression and treatment. J Viral Hepat.

[8] Ginès P, Krag A, Abraldes JG, Solà E, Fabrellas N, Kamath PS (2021). Liver cirrhosis. Lancet.

[9] Heath RD, Mir F, Ibdah JA, Tahan V (2016). Microbiome alterations observed in liver diseases present opportunities for potential fecal transplantation. Turk J Gastroenterol.

[10] Cheng YW, Alhaffar D, Saha S (2021). Fecal microbiota transplantation is safe and effective in patients with Clostridioides difficile infection and cirrhosis. Clin Gastroenterol Hepatol.

[11] Kao D, Roach B, Park H, Hotte N, Madsen K, Bain V, Tandon P (2016). Fecal microbiota transplantation in the management of hepatic encephalopathy. Hepatology.

[12] Bajaj JS, O'Leary JG, Reddy KR (2012). Second infections independently increase mortality in hospitalized patients with cirrhosis: the North American consortium for the study of end-stage liver disease (NACSELD) experience. Hepatology.

[13] Meighani A, Alimirah M, Ramesh M, Salgia R (2020). Fecal microbiota transplantation for Clostridioides difficile infection in patients with chronic liver disease. Int J Hepatol.

[14] Bloom PP, Tapper EB, Young VB, Lok AS (2021). Microbiome therapeutics for hepatic encephalopathy. J Hepatol.

[15] Mullish BH, Quraishi MN, Segal JP, Williams HR, Goldenberg SD (2018). Introduction to the joint British Society of Gastroenterology (BSG) and Healthcare Infection Society (HIS) faecal microbiota transplant guidelines. J Hosp Infect.

[16] Debast SB, Bauer MP, Kuijper EJ (2014). European society of clinical microbiology and infectious diseases: update of the treatment guidance document for Clostridium difficile infection. Clin Microbiol Infect.

[17] American College of Cardiology/American Heart Association Task Force on Practice Guidelines, Obesity Expert Panel, 2013 2013 (2014). Expert panel report: guidelines (2013) for the management of overweight and obesity in adults. Obesity (Silver Spring).

[18] Bajaj JS, Gavis EA, Fagan A (2021). A randomized clinical trial of fecal microbiota transplant for alcohol use disorder. Hepatology.

[19] Li SW, Wang K, Yu YQ, Wang HB, Li YH, Xu JM (2013). Psychometric hepatic encephalopathy score for diagnosis of minimal hepatic encephalopathy in China. World J Gastroenterol.

[20] Cunha-Silva M, Neto FL, de Araújo PS (2022). EncephalApp Stroop test validation for the screening of minimal hepatic encephalopathy in Brazil. Ann Hepatol.

[21] Weissenborn K, Ennen JC, Schomerus H, Rückert N, Hecker H (2001). Neuropsychological characterization of hepatic encephalopathy. J Hepatol.

[22] Pringle PL, Soto MT, Chung RT, Hohmann E (2019). Patients with cirrhosis require more fecal microbiota capsules to cure refractory and recurrent Clostridium difficile infections. Clin Gastroenterol Hepatol.

[23] Hussain S, Khan MS, Jamali MC, Siddiqui AN, Gupta G, Hussain MS, Husain FM (2021). Impact of bariatric surgery in reducing macrovascular complications in severely obese T2DM patients. Obes Surg.

[24] Bajaj JS, Salzman NH, Acharya C (2019). Fecal microbial transplant capsules are safe in hepatic encephalopathy: a phase 1, randomized, placebo-controlled trial. Hepatology.

[25] Mehta R, Kabrawala M, Nandwani S, Kalra P, Patel C, Desai P, Parekh K (2018). Preliminary experience with single fecal microbiota transplant for treatment of recurrent overt hepatic encephalopathy-a case series. Indian J Gastroenterol.

[26] Olmedo M, Reigadas E, Valerio M (2019). Is it reasonable to perform fecal microbiota transplantation for recurrent Clostridium difficile infection in patients with liver cirrhosis?. Rev Esp Quimioter.

[27] Shogbesan O, Poudel DR, Victor S, Jehangir A, Fadahunsi O, Shogbesan G, Donato A (2018). A systematic review of the efficacy and safety of fecal microbiota transplant for Clostridium difficile infection in immunocompromised patients. Can J Gastroenterol Hepatol.

[28] Youngster I, Sauk J, Pindar C (2014). Fecal microbiota transplant for relapsing Clostridium difficile infection using a frozen inoculum from unrelated donors: a randomized, open-label, controlled pilot study. Clin Infect Dis.

[29] Youngster I, Russell GH, Pindar C, Ziv-Baran T, Sauk J, Hohmann EL (2014). Oral, capsulized, frozen fecal microbiota transplantation for relapsing Clostridium difficile infection. JAMA.

[30] Bajaj JS, Shamsaddini A, Fagan A (2021). Distinct gut microbial compositional and functional changes associated with impaired inhibitory control in patients with cirrhosis. Gut Microbes.

[31] Seong H, Lee SK, Cheon JH (2020). Fecal microbiota transplantation for multidrug-resistant organism: efficacy and response prediction. J Infect.

[32] Fischer M, Kao D, Mehta SR (2016). Predictors of early failure after fecal microbiota transplantation for the therapy of Clostridium difficile infection: a multicenter study. Am J Gastroenterol.

[33] Allegretti JR, Kao D, Phelps E (2019). Risk of Clostridium difficile infection with systemic antimicrobial therapy following successful fecal microbiota transplant: should we recommend anti-Clostridium difficile antibiotic prophylaxis?. Dig Dis Sci.

[34] Suez J, Zmora N, Zilberman-Schapira G (2018). Post-antibiotic gut mucosal microbiome reconstitution is impaired by probiotics and improved by autologous FMT. Cell.

